# REVIEW OF WORKPLACE-RELATED GUIDANCE PRESENTED BY ORGANIZATIONS DEALING WITH DEMENTIA OR MILD COGNITIVE IMPAIRMENT

**DOI:** 10.1093/geroni/igac059.495

**Published:** 2022-12-20

**Authors:** James Carino, Philip Taylor, Damian Morgan

**Affiliations:** Federation University Australia, Victoria, Victoria, Australia; Federation University Australia, Victoria, Victoria, Australia; James Cook University, Townsville, Queensland, Australia

## Abstract

A range of organisations in English-speaking countries have prepared guidance materials for individuals who are facing concerns regarding dementia while employed. Some of these organisations have also prepared guidance materials for employers to assist them in engaging employees facing this condition. These are available on organisation websites and may be presented as a downloadable booklet, guide or one or more web pages, aiming to inform about the issues that need to be considered. Information of this type is becoming more important in assisting both parties as dementia is being detected and diagnosed earlier, and individuals need to consider their options regarding their future and their ability to continue to work. This presentation reports on a systematic analysis of around guidance items appearing on these websites. The topic areas, content, and approaches used to present this information to its intended audience were reviewed and compared. Analysis showed important gaps in content coverage from one guidance to another. No guidance presented by any of these organisations covers all areas identified that may potentially interest either employees or employers and in most cases it is not presented coherently for these groups. Employee topics that would benefit from further development include awareness of the condition, the importance of diagnosis and support, use of technology to assist in continuing to be functional and productive, and steps in transition from work to other activities. Employer topics that could be developed further include building awareness amongst employers and the workforce, identifying dementia in the workplace, engaging the wider work group, and positively managing employee separation from work.

